# A comparative study on the overlapping effects of clinically applicable therapeutic interventions in patients with central nervous system damage

**DOI:** 10.1515/med-2023-0828

**Published:** 2023-10-27

**Authors:** Jung-Ho Lee, Dae-Hwan Lee

**Affiliations:** Physical therapy, Kyungdong University, Goseong-gun, Republic of Korea

**Keywords:** overlapping effects, stroke, rehabilitation, feedback

## Abstract

This study was conducted to investigate the effects of anti-gravity treadmill (AGT) training, which provides visual feedback and Biorescue training on proprioception, muscle strength, balance, and gait, in stroke patients. A total of 45 people diagnosed with post-stroke were included as study subjects; they were randomized to an AGT training group provided with visual feedback (Group A), a Biorescue training group provided with visual feedback (Group B), and an AGT/Biorescue group that subsequently received AGT training and Biorescue training (Group C). A muscle strength-measuring device was used to evaluate muscle strength. Timed Up and Go and Bug Balance Scale assessment sheets were used to evaluate balance ability. Dartfish software was used to evaluate gait ability. The results of the study showed that Groups A and C had a significant increase in muscle strength compared with Group B; in terms of balance and gait abilities, Group C showed a significant increase in balance ability and gait speed and a significant change in knee joint angle compared with Groups A and B. In conclusion, this study suggests that including a method that applies multiple therapeutic interventions is desirable in the rehabilitation of stroke patients to improve their independence.

## 1 Introduction

Stroke is caused either by cerebral infarction, in which a blood vessel in the brain is blocked by cerebral hemorrhage, in which blood is drained into substantial structures in the brain due to a ruptured blood vessel [1]. Stroke symptoms include not only sensory, motor, and cognitive impairments but also decreased coordination, balance, and muscle tone control. Sensory motor disorders affect muscle strength, balance, and normal gait, which increase dependence on daily living activities; thus, long-term rehabilitation is required. As dependence on daily life increases or the rehabilitation period is extended, the burden on medical expenses increases. Therefore, much attention is currently being given to rehabilitation to achieve independent walking, which plays the most critical part in reducing dependence on daily life [2,3].

Restoration of balance and gait abilities in stroke patients is considered the most basic element for improving daily living activities and enabling independent activities through functional recovery. Therefore, the common goal of rehabilitation in stroke patients with these problems is to reeducate functional performance and improve gait and balance, aiming to overcome the limit of instability to restore independence [4]. At present, the most applied general therapeutic approaches to restore balance and gait in stroke patients are functional electrical stimulation therapy, treadmill training, the Bobath approach, proprioceptive neuromuscular facilitation, the Brunnstrom approach, and stabilization exercises. These approaches are regarded as treatments that improve functional recovery based on neurophysiological theories [5,6,7].

Muscle weakness occurs after a stroke, and muscle weakness in a patient indicates severe damage. Muscle weakness after stroke may be caused by a decrease in muscle fiber size and excitation rate, atrophy of type 2 muscle fibers, increased fatigue, a decrease in the number of motor units, or changes in motor unit recruitment [8]. Clinically, muscle weakness is a factor that limits the functional rehabilitation of stroke patients, and the measurement of muscle strength is a crucial item for predicting gait ability. Given that muscle weakness decreases gait speed and endurance, which makes tools or other people’s help during walking necessary for the patient and causes difficulty in changing their posture independently, enhancing the strength of the impaired muscle is the goal of therapeutic intervention to enable the patient to perform functional movements [9].

Stroke patients experience difficulty in controlling their movements due to muscle weakness, abnormal muscle tension, and abnormal movement patterns [10]. Stroke patients tend to use only the unaffected limb rather than the paralyzed limb for an extended period; thus, they often experience muscle weakness in the upper and lower limbs on the paralyzed side [11]. Due to muscle weakness in the upper and lower extremities on the paralyzed side, approximately 61–80% of the total body weight of stroke patients is likely to shift toward the lower extremity on the unaffected side in the standing position. Consequently, they show asymmetric body balance and defects in gait, weight transfer, and voluntary movement. Furthermore, since the center of gravity of the body of stroke patients shifts to the unaffected side, symmetrical weight transfer barely occurs in response to external shaking, reducing their ability to maintain balance. Furthermore, when postural agitation occurs, abnormal muscle recruitment arises, and endurance required for weight bearing is also reduced, making it increasingly difficult for stroke patients to maintain their posture [12,13].

The main cause of the decrease in gait speed and endurance in stroke patients is muscle weakness [10]. Progressive resistance training is a training method that enhances muscle strength by gradually increasing the resistance applied to the muscle. This method is effective in strengthening muscles on both the affected and unaffected sides of stroke patients and in recovering gait function by increasing the muscle strength of the lower extremities [14]. Systematic research on these studies has demonstrated that progressive resistance training is effective in increasing muscle strength after stroke. The key elements of progressive resistance training are the supply of sufficient resistance and the application of a training program over a sufficient period (at least 4 weeks) for the gradual increase in resistance and muscle strength. In addition, various muscle strengthening programs using other clinical intervention methods, such as elastic bands, weight training equipment, and isokinetic equipment, are utilized to overcome the muscle weakness problem in stroke patients [15].

In the posture of a stroke patient, the control ability of the central nervous system plays a significant role in maintaining the center of the body within the stability limit. Stroke patients with a damaged central nervous system lose their motor control ability, which maintains their center of gravity, leading to asymmetrical weight support and consequently shifting 60–90% of their body weight toward the unaffected side [16]. This unbalanced weight shift has direct effects, such as unstable gait and reduced gait speed; it also increases the risk of falls – approximately 25% of stroke patients experience falls. Therefore, postural control that enables well-balanced body weight support on both sides is an important goal of rehabilitation training for stroke patients to secure independence in their daily lives [17].

Gait and balance abilities are measures that can determine the recovery level of stroke patients and are thus used as important indicators necessary for independent living [12,13]. Due to their decreased gait ability, stroke patients commonly show a unilateral gait pattern, asymmetric gait length, shortened single-leg support time, decreased motion of the hip and knee joints due to abnormal muscle tension, and increased plantar flexion of the foot [18]. Deterioration of balance ability in stroke patients is caused by reduced basic input sense and difficulty in integrating the input sense. In addition, stroke patients experience difficulties in maintaining balance because they lack postural stability due to their reduced motor control ability and weakened muscle strength. They also show asymmetric weight support and increased postural sway while standing due to their lost balance sense [19].

Since stroke patients have physical function impairments, they exhibit more asymmetric patterns between the paralyzed side and the unaffected side in many areas, such as stride length, step length, step speed, stance phase, and swing phase, compared with the general population at the same age [9]. Although 75% of stroke patients can walk, 50% experience walking disabilities. Gait impairment is caused by a decrease in balance ability due to reduced muscle control ability and proprioception and difficulty in weight transfer. In contrast to healthy people, stroke patients show reduced mobility and stability, reduced gait speed, increased energy inefficiency, altered gait patterns, and reduced restrictive walking endurance [20]. Stroke patients are unable to distribute their total body weight evenly over both legs. Independent gait ability is one of the most crucial factors in the quality of life and participation in daily life and is one of the most important abilities that must be improved through physical therapy to help patients return to their daily lives [21].

Various approaches have been studied to improve the gait ability of patients with post-stroke hemiplegia [13,14,22]. In addition, in stroke patients, if they walk slowly by reducing their gait speed, the one-sided leaning of the gait becomes much more severe. This is because increased gait speed activates the central tendency generator in the spinal cord or brainstem, which improves gait ability. Based on this gait control ability of the central tendency generator, treadmill exercise is used as a therapeutic intervention method for the rehabilitation of patients with damaged central nervous system [23,24].

In a comparison between gait training conducted on normal ground and gait training on a treadmill, gait training using a treadmill is more effective; thus, the importance of gait training using a treadmill is increasing [23]. Recently, gait training on a treadmill in a partial weight support condition, which is based on task-oriented training, has been reported to aid in restoring the gait ability of stroke patients through muscle strengthening, lower body stabilization, and reeducation of balance and gait pattern control. When a safety assist device is worn during gait training on a treadmill, the abnormal gait pattern is improved, and stride length and speed are increased [25]. In addition, an anti-gravity treadmill (AGT) enables weight-bearing exercise in the early stage after surgery; during the early weight-bearing exercise, the training can be performed by adjusting the patient’s weight in 1% increments/decrements and by controlling pain and adjusting exercise intensity. In addition, an advantage of this training method is that a safety device prevents falls and slips during exercise; thus, exercise can be safely performed [26].

After a stroke, many patients suffer from loss of visual information with impaired proprioception, which results in loss of visual and spatial perception [27]. Feedback training is used to improve the balance and walking ability of stroke patients. In visual feedback training, the actual movement of the subject is reflected on the screen in various forms to imitate the actual movement, informing the subject of their position in the virtual space or whether the movement was successful using visual elements. In this manner, this technique provides biofeedback for the subject’s movement toward them [28].

Although numerous studies regarding the efficient rehabilitation of stroke patients have been conducted [20], research on training methods using visual biofeedback is lacking. Most studies focusing on the fragmentary physical elements of stroke patients have been conducted. In addition, studies on the effects of rehabilitation therapy caused by overlapping treatment methods are insufficient. Therefore, this study investigated the effects of treatment methods accompanying biofeedback on muscle strength, balance, and gait ability, which are key factors that indicate the level of rehabilitation in stroke patients, using an AGT and a Biorescue system that can provide visual feedback. In addition, the overlapping effect of the two treatments that provide feedback was investigated by sequentially applying the AGT and Biorescue systems.

## 2 Materials and methods

### 2.1 Subjects

A total of 45 patients diagnosed with post-stroke hemiplegia based on magnetic resonance imaging by a rehabilitation medicine specialist at a rehabilitation hospital were included in this study as study subjects. Before participating in this study, all patients were provided with an explanation of the overall purpose, process, risks, and side effects of the study, and they then completed the informed consent form. All research procedures were performed under the supervision of the Institutional Review Board in accordance with the Declaration of Helsinki. All experimental procedures and protocols have been approved by the research ethics committee following the guidelines of the university.

### 2.2 Design

The study subjects were divided into Group A, which performed the AGT training with visual feedback; Group B, which trained using the Biorescue system with visual feedback; and Group C, which sequentially performed AGT training and Biorescue training to examine the overlapping effects of the treatment. A total of 15 participants were assigned to each group through randomization.

1. The inclusion criteria for the subjects in this study were as follows: (A) Those who were diagnosed more than 6 months ago with hemiplegic stroke using a brain imaging-based specialist. (B) Those without any cognitive impairment that interfered with participation in the study, that is, those with a mini-mental state examination score of 24 points or higher. (C) Those who can walk more than 10 m independently using a walking aid or under the supervision of a therapist. (D) Those who were diagnosed with defects in balance and gait by a specialist. (E) Those without any visual impairment, problems in the cardio-respiratory system, or other neurological problems that may affect walking. (F) Those who agreed to participate in the study after being informed about the purpose and method of this study.
2. The exclusion criteria for the subjects in this study were as follows: (A) Those with recurrent strokes. (B) Those with neurological or orthopedic disorders other than stroke. (C) Those who withdrew their intention to participate in the study.

### 2.3 Intervention

AGT can adjust the load applied to the knee from 20 to 90% in 1% increments/decrements, enabling walking exercises without pain. Furthermore, by connecting the patient wearing a suit exclusively designed for an AGT with the AGT, the risk of injury from losing balance and falling during exercise is minimized. Therefore, AGT is a device that enables research to be conducted safely. The exercise program used in this study was performed according to the protocol suggested by the manufacturer of Alter-G (Alter-G^®^; AlterG, Inc., Fremont, CA, USA) using the AGT equipment after some modifications and supplementations. It was performed three times a week for 30 min for over 2 weeks with a physical therapist with professional knowledge of neurological physical therapies and more than 10 years of experience.

In this study, for the initial treatment using the AGT exercise program, weight was reduced by up to 80%, and the speed was set to 0.5 km/h. The weight load and speed were then gradually increased. The exercise program was divided into three stages. In the first stage (weeks 1–4), the weight load was gradually increased by 1%, from 20 to 30% as the program progressed, and the speed was maintained at 0.5 km/h in all stages. In the second stage (weeks 5–8), the goal was set to 30–50%, and the weight load was increased by 1 or 2% depending on the program and progressed to 50% in the final week. In the third and final stage (weeks 5–8), the weight load was raised from 1 to 4% according to the patient’s adaptation level, so that a weight load of 80% or more was applied in the final week.

In this study, cognitive tasks and balance training were performed using the Biorescue (RM INGENIERIRE, Rodez, France) equipment. The subjects participated in the experiment while standing on a decompression platform 1.5 m away from the monitor, and they performed the tasks provided by each program by moving their bodies. Before the application of the exercise, all subjects were explained how to use the equipment and precautions. In this study, weight transfer training using trunk movements (i.e., flexion, extension, lateral flexion, and rotation), upper and lower extremity movement, and cognitive task training were performed simultaneously. In this study, both training methods were performed for 15 min each. The first training was to memorize four card pictures located in four directions (east, west, south, and north), and a card picture with the same image as in the presented card picture was searched by shifting the weight. The second training was training to find the same card picture as in the flipped card picture through a shift in weight among eight card pictures with two identical card pictures. Initially, the exercise intensity was set according to each subject, and the intensity gradually increased. Safety bars were installed on the front and sides to prevent falls during training, and a physical therapist with more than 10 years of clinical experience and 10 years of experience in instructing Biorescue training supervised and instructed the patient during the training. In addition, one assistant helped the subject with training to secure stability. Every training was applied for 30 min each time, three times a week for a total of 12 weeks.

### 2.4 Outcome measure

#### 2.4.1 Muscle strength of lower extremities

In this study, lower extremity muscle strength was evaluated by measuring the muscle strength of the knee extensor and flexor on the affected side using a manual muscle strength measuring device. In this study, the pressure at the maximum isometric contraction of each muscle was measured three times, and the average value was used for the analysis. Knee joint extension and flexion were measured in a sitting position. To measure the knee joint extensor muscle, we instructed the subject to straighten the knee with a pressure plate of the manual muscle strength measuring device placed on the front side of the ankle. To measure the knee joint flexor muscle, we instructed the subject to bend the knee with a pressure plate placed on the heel.

#### 2.4.2 Static and dynamic balance

Timed Up and Go (TUG) test: The TUG is used to measure mobility, dynamic balance, and moving ability and is particularly useful for evaluating gait and the ability to perform the task of turning around obstacles and then returning to the starting point. This method measures the time it takes for a round trip, starting from sitting on a chair that is 50 cm high, going around the turning point that is 3 m away, and returning and sitting back on the chair. This test is an evaluation tool developed to examine functional mobility. It is an easy, simple, and fast test method that can be performed rapidly because mobility can be quantified; thus, it is currently widely used in clinical practice. It is also an evaluation method that can predict the risk of falls by assessing the ability to maintain balance while walking in a short time. If the result of TUG is less than 10 s, no problem with gait ability is considered; if the result is from 11 to 20 s, a problem with gait ability is considered; if it takes more than 20 s, functional motor impairment is considered. In this study, the subjects were allowed to make one round trip, and the time to take the next three round trips was then measured. The average time was recorded as the measurement for the analysis.

The Bug Balance Scale (BBS) is a tool designed to evaluate balance ability. Currently, it is widely used clinically to determine the degree of functional performance about balance in stroke patients and has the advantage of easy evaluation, regardless of location. The BBS is a tool used to measure functional standing balance, consisting of a total of 14 items in three areas: sitting, standing posture, and postural change. It has a total of 14 items on a 5-point scale, from a minimum of 0 to a maximum of 4 points, with a total of 56 points. The higher the score, the better the state of balance ability. In general, 0–20 points indicate a need to use a wheelchair, and 21–40 points indicate a need for walking with an aid or assistance. For complete independent walking, 41 points or higher are required, and 45 points or higher are required for independent and safe movement. Recently, BBS has also been used to evaluate the balance ability of patients with stroke, cerebral palsy, and Parkinson’s disease.

#### 2.4.3 Gait ability

This study analyzed the patient’s gait using the Dartfish Express PC version 10.0 (DFKOREA, Korea). Dartfish is a software that can analyze and interpret motions by taking videos using a camera; it is widely used in the field of rehabilitation. In this study, to measure and compare the changes in the time point at which the knee angle becomes the greatest in the swing phase during walking, we fixed the camera 3 m away from the 10 m path, where the subject walks on a flat surface in the laboratory. The anatomical locations, such as the greater trochanter of the femur, the lateral epicondyle of the femur, and the lateral malleolus, were attached with 3 cm markers. When the subject was walking, the angle at which the extension lines connected with each marker met was measured, and the angle of the knee joint during the mid-swing phase was recorded and then measured using the Dartfish software program for motion analysis. This procedure was repeated three times. The average value was then used for the analysis.

### 2.5 Statistical analysis

In this study, IBM SPSS Statistics 20.0 was used for statistical processing. The mean and standard deviation of each variable, which was measured to evaluate muscle strength, balance, and gait, were calculated through descriptive statistics, and their normality was examined through the Kolmogorov–Smirnov test. A paired-sample *t*-test was used to compare the within-group differences between the before and after training for muscle strength, balance, and gait. An analysis of variance was performed to compare the difference in treatment effects between groups, and LSD was used as a post hoc test. The statistical significance level (*α*) was set to 0.05 or less.

## 3 Results

The study subjects’ general characteristics and pre-test assessments, including knee extensor, knee flexor, TUG, BBS, and knee joint angle, were homogenous with no statistically significant differences (Table 1).

**Table 1:** General characteristics of subjects and homogeneity test of pre-test assessments

	Group A	Group B	Group C	*P*
Age (years)	65.84 ± 4.22	67.09 ± 6.13	66.54 ± 5.08	0.512
Weight (kg)	56.18 ± 6.81	53.63 ± 5.08	57.34 ± 6.42	0.264
Knee extensor (kg)	8.15 ± 2.95	7.64 ± 3.78	8.22 ± 3.05	0.763
Knee flexor (kg)	4.11 ± 1.87	4.98 ± 2.14	3.98 ± 1.57	0.864
TUG (s)	30.48 ± 9.56	28.46 ± 10.68	27.67 ± 9.37	0.184
BBS (score)	28.64 ± 5.69	27.61 ± 4.55	30.14 ± 7.01	0.149
Knee joint (angle)	30.15 ± 8.31	28.14 ± 8.21	31.88 ± 9.17	0.138

Group A, anti-gravity treadmill training group provided with visual feedback; Group B, Biorescue training group provided with visual feedback; Group C, anti-gravity treadmill training + Biorescue training group provided with visual feedback; TUG: Timed Up and Go; BBS: Bug Balance Scale.

In the comparison of the within-group differences between the before and after training for knee extensor muscle strength, a statistically significant increase in muscle strength was observed in Group A with AGT training and Group C with AGT training, followed by Biorescue training (Table 2). A significant difference was found in the analysis of variance, which was conducted to determine the difference in the treatment effect between groups; thus, a post hoc test was performed. Groups A and C showed a greater increase in extensor muscle strength than Group B, and no statistically significant difference was observed in the amount of increase between Groups A and C.

**Table 2:** Comparison of muscle strength of the knee extensors on the paretic side

Extensors	Group A	Group B	Group C	*f*	*P*
Pre-test	8.15 ± 2.95	7.64 ± 3.78	8.22 ± 3.05	0.619	0.763
Post-test	11.37 ± 3.67	8.95 ± 2.64	13.46 ± 4.12	4.684	0.000*
*t*	−2.05	−0.98	−4.57		
*P*	0.024*	0.059	0.000*		

Group A, anti-gravity treadmill training group provided with visual feedback; Group B, Biorescue training group provided with visual feedback; Group C, anti-gravity treadmill training + Biorescue training group provided with visual feedback; unit, ㎏; *P < 0.05.

Likewise, in the comparison of the within-group differences between the before and after training for the knee flexor, a statistically significant increase in muscle strength was observed in Group A with AGT training and Group C with AGT training, followed by Biorescue training. Group B showed an increase in the mean value; however, it was not statistically significant (Table 3). A significant difference was found in the analysis of variance, which was conducted to determine the inter-group difference in the treatment effect; thus, a post hoc test was performed. Groups A and C showed a greater increase in flexor muscle strength than Group B, and no statistically significant difference was observed between Groups A and C.

**Table 3:** Comparison of muscle strength of the knee flexors on the paretic side

Flexors	Group A	Group B	Group C	*f*	*P*
Pre-test	4.11 ± 1.87	4.98 ± 2.14	3.98 ± 1.57	0.538	0.864
Post-test	6.78 ± 2.06	5.39 ± 1.98	7.44 ± 2.24	3.612	0.048*
*t*	−0.26	−0.53	−3.87		
*P*	0.018*	0.071	0.008*		

Group A, anti-gravity treadmill training group provided with visual feedback; Group B, Biorescue training group provided with visual feedback; Group C, anti-gravity treadmill training + Biorescue training group provided with visual feedback; unit, ㎏; *P < 0.05.

In the comparison of the within-group differences between the before and after training for TUG, a statistically significant increase in gait speed in all three groups was found: Group A with AGT training, Group B with Biorescue training, and Group C with AGT training, followed by Biorescue training (Table 4). A significant difference was found in the analysis of variance, which was conducted to determine the inter-group difference in the treatment effect; thus, a post hoc test was performed. Groups A and C showed a greater increase in gait speed than Group B, and Group C showed a statistically significant difference in the increase in gait speed compared with Group A.

**Table 4:** Comparison of Timed Up and Go test

TUG	Group A	Group B	Group C	*f*	*P*
Pre-test	30.48 ± 9.56	28.46 ± 10.68	27.67 ± 9.37	2.267	0.184
Post-test	26.68 ± 10.25	26.55 ± 9.27	21.82 ± 8.95	5.591	0.000*
*t*	3.71	1.96	5.33		
*P*	0.000*	0.027*	0.000*		

Group A, anti-gravity treadmill training group provided with visual feedback; Group B, Biorescue training group provided with visual feedback; Group C, anti-gravity treadmill training + Biorescue training group provided with visual feedback; unit, seconds; *P < 0.05.

In the comparison of the within-group differences between the before and after training for BBS, a statistically significant increase in the BBS score was observed in Group A with AGT training and Group C with AGT training, followed by Biorescue training. Group B showed an increase in the mean value; however, it was not statistically significant (Table 5). A significant difference was found in the analysis of variance, which was conducted to determine the inter-group difference in the treatment effect; thus, a post hoc test was performed. Groups A and C showed a greater increase in the BBS score than Group B, and Group C showed a statistically significant difference in the amount of increase compared with Group A.

**Table 5:** Comparison of BBS

BBS	Group A	Group B	Group C	*f*	*P*
Pre-test	28.64 ± 5.69	27.61 ± 4.55	30.14 ± 7.01	2.687	0.149
Post-test	32.36 ± 6.37	29.45 ± 6.87	36.43 ± 5.23	5.132	0.000*
*T*	−4.03	−1.08	−6.12		
*P*	0.000*	0.053	0.000*		

Group A, anti-gravity treadmill training group provided with visual feedback; Group B, Biorescue training group provided with visual feedback; Group C, anti-gravity treadmill training + Biorescue training group provided with visual feedback; unit, score; *P < 0.05.

In the comparison of the within-group differences between the before and after training for the change in knee angle during walking, a statistically significant change was observed in the knee angle in all three groups: Group A with AGT training, Group B with Biorescue training, and Group C with AGT training, followed by Biorescue training (Table 6). A significant difference was found in the analysis of variance, which was conducted to determine the inter-group difference in the treatment effect; thus, a post hoc test was performed. Groups A and C showed a greater change in knee angle during walking than Group B, and Group C showed a statistically significant change in knee angle compared with Group A.

**Table 6:** Comparison of the angle of the knee joint

Knee angle	Group A	Group B	Group C	*f*	*P*
Pre-test	30.15 ± 8.31	28.14 ± 8.21	31.88 ± 9.17	2.857	0.138
Post-test	33.67 ± 9.03	29.66 ± 7.66	37.54 ± 8.34	5.984	0.000*
*t*	−3.78	−1.35	−6.03		
*P*	0.000*	0.041*	0.000*		

Group A, anti-gravity treadmill training group provided with visual feedback; Group B, Biorescue training group provided with visual feedback; Group C, anti-gravity treadmill training + Biorescue training group provided with visual feedback; unit, angle; *P < 0.05.

## 4 Discussion

Neuroplasticity is a phenomenon in which the brain nerves change and reorganize their structures and functions by learning from the experience of external stimuli, and it occurs continuously throughout life. During periods of vigorous learning of other languages or motor skills, such as during childhood, the activity of new neural pathways is maximized [29]. Although the potential decreases in adulthood or old age, a certain level of neuroplasticity is maintained throughout life. The brain is composed of nerve cells (neurons) and glial cells that are connected. Learning can occur through changes in the length of nerve cell connections, the addition or removal of connections, and the formation of new nerve cells, and neuroplasticity is related to such a learning process [30].

Neuroplasticity, the process by which the human brain changes according to experience also refers to the ability of the brain to reorganize or rearrange after damage to the central nervous system. It can be considered an adaptation process of the nervous system in which the function and shape of the cerebral cortex change to suit the surrounding environment or lesion [31]. Brain plasticity plays a significant role in the recovery of cerebral functions, such as learning and memory, after a brain lesion occurs. Plastic changes mainly occur in synapses widely distributed in the cerebrum, through which the network in the brain is reorganized [32]. In addition, recent studies have emphasized the importance of afferent sensory information in the coordination between limbs. As reported, the integration of sensory movements that occur between the left and right cerebral hemispheres is a crucial factor in the recovery of motor functions after stroke, and the rehabilitation strategy that induces active use of sensory feedback is effective. When such sensory information is transmitted to the sensory center area, it can be involved in the control of voluntary movements related to the main motor area adjacent to the sensory center area, which supports the effectiveness of rehabilitation treatment using various sensory stimuli [33,34]. In summary, the learning of new motor skills by the normal central nervous system and the reacquisition of functional activity after a brain injury are similar in many aspects. The biomechanical changes that occur when skills are reacquired after a stroke in the motor performance stage are similar to those that occur when people without a lesion learn new skills [35].

Stroke-related hemiplegia patients have a decreased sensation in each part of the body on the paralyzed side, with lowered abilities to move their limbs and interact with the environment due to the decrease in proprioception, which plays a key role in postural sway control in various environments [12]. In terms of proprioception, action potentials generated from proprioceptive sensory receptors, such as muscles, joints, and tendons, act afferently, which makes the nerve center sense and feel the body’s movement and positional sense, thereby enabling the body to control balance and posture automatically according to changes in various external environments and situations. Proprioception is a sense that includes a positional sense that informs the position of each joint and a motor sense that detects the degree and speed of muscle movement. Motor sense refers to the ability to perceive the direction of movement and responses [28,36].

As a crucial factor in the performance of functional activities, proprioceptive sense has a significant correlation with the ability to maintain posture; thus, it is used as a predictor of various injuries [37]. It also can recognize joint angles using the positional sense, which provides the central nervous system with information on the recognition of body position and posture, the angles and angular velocity of all joints involved in the movement in each movement plane, and the ratio of their movements. This sense plays the most important role in maintaining dynamic joint stability, inducing normal movement, and safely protecting joints from external damage [38]. In addition, the sense of movement or position of joints is affected by various types of senses, including visual and skin senses; loss of positional sense leads to lowered motor performance and difficulties in performing activities in daily life. Therefore, proprioception is a conscious sense of joint motion and position, and it has been used as an important factor in observing the prognosis for the rehabilitation of a patient [39].

Stroke patients experience a lot of difficulties in walking due to the impairment of proprioception that occurs after damage to the central nervous system. Most stroke patients experience difficulties in walking because of the ground environment or exposed locational factors due to not only muscle weakness, abnormal muscle movement, stiffness, and decreased balance ability but also decreased proprioception that interferes with normal walking [9,12]. Disabilities appear in the legs due to neurological damage, and these disabilities can cause asymmetric gait and lowered balance ability. In addition, as a result of asymmetric gait, abnormal gait patterns, such as slow gait speed, the occurrence of instability during gait, differences in stride length during gait, short stance phase on the paralyzed side, and relatively long swing phase, appear [40]. For stroke patients, a lack of walking ability is considered the main cause that limits their daily living activities in the early stages after the onset of the disease.

Balance is the ability to control the position of joints or muscle activity to maintain body weight on the supporting ground, and postural stability means not losing balance while standing or not falling while performing dynamic activities [4]. Balance can also be divided into static balance and dynamic balance. Static balance refers to the ability to stand on a fixed support surface without shaking, and dynamic balance refers to balance while moving on a support surface, with an external stimulus or while moving unaided [16]. Balance disorders that occur after stroke appear due to changes in the motor control sense and the integration thereof. Balance ability-related problems occur in the sub-acute phase after the first onset of stroke, and trunk damage, balance limitations, and postural control disorders increase the risk of falls and cause gait disturbances in stroke patients. These problems increase the dependence of stroke patients on activities in daily life and reduce their quality of life [41].

Re-establishing the balance function of patients with stroke-related hemiplegia through rehabilitation treatment is an important goal of rehabilitation, and several balance training methods using visual feedback have been studied to improve balance control ability. Providing stroke patients with far more feedback for motor learning than normal people is necessary, and balance retraining of stroke patients using visual feedback improves their balance control ability considerably when feedback is provided. In addition, through visual feedback training, patients can check their positional and postural changes in real time; the patients themselves recognize the postural information and use it to control and maintain their postures [42,43]. A high level of motivation can have a positive effect on the outcomes of rehabilitation training. In this study, visual feedback was provided to all groups to improve balance ability, and improvements in balance ability were observed in the results of the TUG and BBS. Furthermore, a statistically significant increase was observed in balance ability and gait speed in Group C, which received both AGT training and Biorescue training, compared with the other groups. This result can be attributed to the fact that the lower extremity muscle strengthening effect of AGT and the improvement of dynamic and static balance ability through Biorescue training overlapped, creating a remarkable effect.

Stroke patients tend to use only the unaffected limb rather than the paralyzed limb for a long period, which results in muscle weakness in the paralyzed limb. Due to muscle weakness in the lower extremity on the paralyzed side, approximately 60% or more of the total body weight shifts toward the lower extremity on the unaffected side in the standing position. Consequently, stroke patients show asymmetric body balance and defects in gait, weight transfer, and voluntary movement [44,45]. Especially since the center of gravity of the body shifts to the unaffected side in stroke patients, symmetrical weight shift does not occur in response to sudden external disturbances, which reduces their ability to maintain balance and causes abnormal muscle recruitment, making it difficult for them to maintain posture [41].

Muscle weakness is the main cause of a decrease in gait speed in stroke patients; thus, studies on eccentric training have been conducted to find a way to improve muscle strength. Progressive resistance training is a training method that enhances muscle strength by gradually increasing the resistance applied to the muscle. This method is effective in strengthening the muscles on both the affected and unaffected sides of stroke patients [46]. Systematic research on these studies has demonstrated that resistance training is effective in strengthening muscles after stroke. The key elements of progressive resistance training are the supply of sufficient resistance and the application of a training program over a sufficient period (at least 4 weeks) for the gradual increase in resistance and the increase in muscle strength [47]. In this study, an AGT treatment device was used in Groups A and C for progressive resistance training. The extensor and flexor muscles of the knee are important muscles that provide stability and mobility of the knee during walking. In this study, a statistically significant increase in these two muscles was observed in the groups using AGT. This result suggests that the patient’s lower extremity muscle strength increased because the AGT was able to assist the patient in walking repeatedly for 12 days. In addition, this increase in the muscle strength of the lower extremity is likely related to the increase in proprioceptive activity because visual feedback was applied during the AGT training.

The characteristic gait patterns of stroke patients include that while walking, the tip of the toe touches the ground before the heel touches it during the stance phase of the paralyzed side and that the swing phase of the paralyzed side is longer than that of the unaffected side, resulting in asymmetry during walking. Furthermore, compared with the non-paralyzed side, the paralyzed side requires more time for the swing phase during toe-off according to the decrease in the angle change in the knee joint [9,14]. The stance phase of both legs increases, whereas the gait speed and propulsive force decrease, and footdrop and varus are observed in the stance phase on the paralyzed side. This asymmetrical gait decreases gait speed, gait endurance, and balance ability, which limits the independent movement ability of stroke patients. In other words, restoration of gait ability in stroke patients is the goal of rehabilitation that should be considered first to enable them to perform daily living activities independently [16,18].

Compared with healthy people, stroke patients’ activity of the extensor muscle of the knee joint is abnormally higher due to a synergistic pattern over time. This elevated extensor muscle activity is an element necessary for walking; however, excessive extensor muscle activity results in limiting the flexion angle of the knee joint in the swing phase, inducing abnormal compensatory action [48]. Circumduction gait, which is a typical compensatory action, involves walking in a circular motion using the pelvis and hip joint during walking. This causes numerous problems, such as a reduction in gait speed, asymmetrical stride length and time for the stance and swing phases, reduction in the efficiency of muscle activity, and increased energy consumption [49]. In this study, gait was analyzed using the Dartfish program to determine the change in knee flexion angle during walking according to therapeutic interventions. The results of this study showed a statistically significant increase in knee joint angle in all groups in which visual feedback was applied. This result can be attributed to the fact that the sensory information received by the patient through the application of visual feedback during walking continuously stimulated the motor integration center of the brain, followed by the recorrection process, and movement control then occurred. In addition, in Group C, in which both the treadmill training and the Biorescue training that provided visual feedback were applied, a statistically significant flexion of the knee joint was observed compared with the other groups. This result can be attributed to the overlapping effect of the two treatments focusing on visual feedback, muscle strength, and balance ability.

To secure the independence of patients after a stroke, we should apply a treatment method that can simultaneously improve numerous factors, such as proprioception, muscle strength, balance ability, and gait ability, rather than a fragmentary treatment method [20,23]. The findings of this study also showed that new motor control and motor integration can be induced in patients rapidly and easily by applying visual feedback simultaneously during therapeutic interventions. However, this study has limitations: it failed to examine the lasting effect of the treatment since the treatment interventions were applied only for a short period, and it was difficult to generalize the findings of this study because of the small sample size used in this study. Considering that only two fragmentary evaluations were conducted in this study, a further study based on the design and results of this study must be conducted in the future, so that the pattern and duration of the treatment effect and the duration of the overlapping effect can be assessed through continuous application of treatment for more than 6 months and quantitative evaluation every 2 weeks.
